# The Solution Properties of Polymethacrylate Molecular Brushes with Oligo(ethylene glycol) and Oligo(propylene glycol) Side Chains

**DOI:** 10.3390/polym14245556

**Published:** 2022-12-19

**Authors:** Maria Simonova, Alexander Simagin, Denis Kamorin, Sergey Orekhov, Alexander Filippov, Oleg Kazantsev

**Affiliations:** 1 Institute of Macromolecular Compounds of the Russian Academy of Sciences, Bolshoy Prospekt 31, Saint Petersburg 199004, Russia; 2 Research Laboratory “New Polymeric Materials”, Nizhny Novgorod State Technical University n.a. R.E. Alekseev, 24 Minin Street, Nizhny Novgorod 603950, Russia

**Keywords:** methoxy (oligoethylene glycol-block-oligopropylene glycol) methacrylate]s, oligo(ethylene glycol) methacrylates, oligo(propylene glycol) methacrylates, amphiphilic polymers, molecular brushes, radical polymerization, thermoresponsibility

## Abstract

The properties of polymer brushes based on three macromonomers were investigated in aqueous and organic solutions. Methacrylic monomers with different compositions of the oligo(oxyalkylene) substituents and arrangements of the oligo(ethylene glycol) and oligo(propylene glycol) blocks were used for the synthesis of polymers. There were methoxy [oligo(ethylene glycol)_10.3_-block-oligo(propylene glycol)_4.7_] methacrylate, methoxy [oligo(propylene glycol)_8.3_-block-oligo(ethylene glycol)_6.6_] methacrylate, and methoxy oligo(propylene glycol)_4.2_ methacrylate. Molecular brushes were investigated by the methods of molecular hydrodynamics and optics in dilute solutions in acetonitrile, chloroform, and water. The peculiarities of behavior of poly[oligo(oxyalkylene) methacrylates] in aqueous solutions and water-toluene systems have been found; in particular, the solubility of the polymers in water and organic solvents, the polymers equilibrium distribution between the phases, and the surface activity in the water-toluene system have been established. The thermo-responsibility in aqueous solutions and values of a critical concentration of micelle formation were shown. Depending on the arrangement of blocks in the side chains of molecular brushes, they are characterized by different intramolecular density.

## 1. Introduction

Over the past decades, various types of polymer systems, for example, nanogels, polymersomes, micelles, and dendrimers, have been proposed and intensively studied for the controlled delivery of various drugs to diseased organs [1,2,3,4,5,6]. A nanocontainer with a drug encapsulated in it should circulate in the body without causing harmful effects, and only after targeted delivery to the affected intracellular and intratissue area, the drug should leave the container (due to its destruction or due to diffusion from it) and manifest its functional activity without damaging the healthy organs [7].

An important type of nanocontainer is amphiphilic polymer micelles, whose hydrophobic core retains poorly water-soluble drugs, which are then released by diffusion or destruction of micelles under external action. This option is being developed by many researchers and is considered one of the most promising options for drug delivery [8]. There are numerous opportunities to regulate the composition and properties of polymers used for this purpose, which potentially makes it possible to develop polymeric nanocontainers with specified properties for the delivery of a particular drug, taking into account its structure and properties [1].

Micelle formation by block copolymers based on monomers with different hydrophobicity has now been fairly well studied in terms of particularities of micelle formation, morphology, size, and stability. The most widely studied polymers are poly(propylene oxide)s, poly(L-amino acid)s, and poly(ester)s [9]. The review [10] focuses on methods of drug encapsulation into polymeric micelles and the effect of the nature of substituents and length of hydrophobic blocks on encapsulation; the methods of controlled drug release are discussed. Mixed polymeric micelles have been shown to have higher drug-loading capacity, increased circulation time in the blood, and thermodynamic stability.

Bottlebrushes with complex macromolecule architecture offer additional tools for fine-tuning physical properties by varying not only the parameters of the main chain (molecular weight, composition, hydrophilic–hydrophobic balance), but also by working with the chemistry and architecture of the side chains. For example, copolymers with mixed side-chain chemistry have been shown to be effective modifiers for surface and interface properties [11]. It was shown that bottlebrush block copolymers form micelles in aqueous solutions at concentrations 1–2 orders of magnitude lower than their linear counterparts [12] and their CMC is much less sensitive to composition, which makes bottlebrush copolymers more promising for targeted drug delivery in terms of micelle stability under dilution.

Many developed nanocontainers for drug delivery contain polyethylene glycol hydrophilic shells, which provide polymers with good biocompatibility and biodegradability [2,4]. For example, grafted polyethylene glycol fragments are contained in some polymeric means of drug delivery in chemotherapy of oncological diseases [13]. In the last 15 years, oligo(ethylene glycol) methacrylate (OEGM) polymers, which many researchers consider to be more promising than the previously most popular polymers N-isopropyl acrylamide and its analogs, have attracted particular attention from researchers as potential nanocontainers [14,15]. Amphiphilic (co)polymers of OEGM have good biocompatibility, low toxicity, are subject to biodegradation, and—like N-isopropyl acrylamide polymers—can have a lower critical solution temperature (LCST), which is close to the human body temperature. This can further enhance the effectiveness of such polymers for targeted delivery and controlled release of drugs in the body.

The present work proposes the introduction of oligo(propylene glycol) chains into macromolecules, which are much more hydrophobic compared to oligo(ethylene glycol) chains. Amphiphilic polymers containing poly(ethylene glycol) and poly(propylene glycol) blocks are capable of self-organization in aqueous solutions with the formation of micelles with unique properties [16]; their essential feature is also good biocompatibility. Oligo(propylene glycol) fragments can be introduced into polymers as part of methacrylic macromonomers—oligo(propylene glycol) methacrylates (OPGM), but very few studies in this direction have been published. It was shown [17] that the homopolymer of OPGM, containing five oxypropyl units and having an end hydroxyl group, is insoluble in water at room temperature. However, modification of the polymer by adding hydrophilic oligo(ethylene glycol) chains to the end hydroxyl group made it possible to obtain a water-soluble thermoresponsive polymer that forms unimeric micelles [18]. The study of the properties of several polymers having similar compositions has been recently published [19].

Previously, we studied the behavior in solution of homopolymers of monomers with diblock substituent—methoxy [oligo(ethylene glycol)-block-oligo(propylene glycol)] methacrylates —which contained the oligopropylene glycol block located closer to the vinyl group and the oligoethylene glycol block placed at the end of the substituent [20]. In the published article, the length of the oligopropylene glycol block was changing while the length of the oligoethylene glycol block was fixed. It is of interest to obtain information on the influence of the arrangement of the blocks relative to the vinyl group of a monomer. In this study the properties of polymers obtained on the basis of monomers with different arrangement of the blocks relative to the vinyl group were compared.

**Figure 1:**
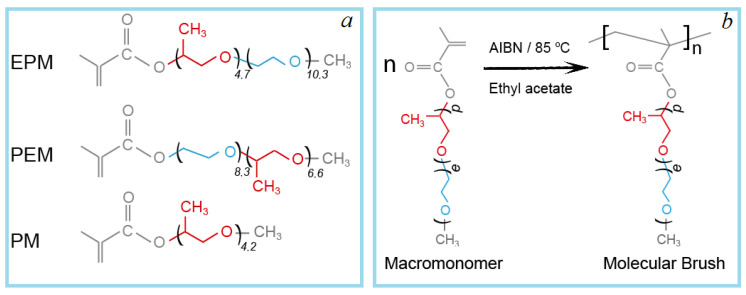
Structures of the macromonomers (**a**) and the scheme of polymerization (**b**).

Three macromonomers were used for the estimation of the influence of the substituent composition: methoxy [oligo(ethylene glycol)_10,3_-block-oligo(propylene glycol)_4,7_] methacrylate (EPM), methoxy [oligo(propylene glycol)_8,3_-block-oligo(ethylene glycol)_6,6_] methacrylate (PEM), and methoxy oligo(propylene glycol)_4,2_ methacrylate (PM). The structures of the macromonomers are shown in Figure 1.

The discussion of the properties of the diblock monomers is of particular interest in comparison with their “monoblock” analogues: oligo(ethylene glycol) methacrylates and oligo(propylene glycol) methacrylates. While oligo(ethylene glycol) methacrylate polymers have been extensively studied and there are many papers describing the behavior of such polymers in water, for poorly water-soluble poly[oligo(propylene glycol) methacrylates], it is considered necessary to include information on the properties. Therefore, the polymer of methoxy oligo(propylene glycol)_4.2_ methacrylate (PM) was obtained, which is interesting to compare with the polymer with the close length of the propylene glycol block, but having also the oligoethylene glycol block (EPM).

## 2. Materials and Methods

Methacrylic acid, p-toluene sulfonic acid, hydroquinone, azobisisobutyronitrile (AIBN), toluene, ethyl acetate, and hexane (Aldrich, Saint Louis, MO, USA) were used in experiments. AIBN was recrystallized twice from ethanol. Organic solvents were used as received. For light scattering experiments, chloroform (density ρ_0_ = 1.486 g∙cm^−3^, dynamic viscosity η_0_ = 0.57 cP and refractive index *n*_0_ = 1.443), tetrahydrofuran (THF, ρ_0_ = 0.890 g∙cm^−3^, η_0_ = 0.46 cP and *n*_0_ = 1.405), acetonitrile (ρ_0_ = 1.486 g∙cm^−3^, η_0_ = 0.57 cP and *n*_0_ = 1.443), and water (ρ_0_ = 1.000 g∙cm^−3^, η_0_ = 0.98 cP and *n*_0_ = 1.333) were used as solvents. For macromonomers synthesis, the previously described [21] method of the esterification of methacrylic acid with methoxy oligo(alkylene glycol)s was carried out at temperature of 120–125 °C in 30 wt% toluene solution in the presence of 2 wt% of p-toluene sulfonic acid as a catalyst and 0.3 wt% of hydroquinone as a polymerization inhibitor. The monomers yield was 80–85%, with purity of 93.0–98.0%. Previous to polymerization, the macromonomers were passed through a basic alumina column to remove inhibitors. PEM: ^1^H NMR [400 MHz, DMSO-D6, 25 °C, δ = 2.5 (DMSO)]: δ = 6.03 1H (C**H**_2_=), δ = 5.69 1H (C**H**_2_=), 4.21 2H (COOC**H**_2_-), δ = 3.65–3.29 55H (-C**H**_2_O(C**H**_2_C**H**_2_O)_n_(C**H_2_**C**H**(CH_3_)O)_m_-), δ = 3.25 3H (-OC**H**_3_), δ = 1.88 3H (CH_2_=C(C**H**_3_)COO-), δ = 1.05 18H (-(CH_2_CH(C**H**_3_)O)_m_-).PM: ^1^H NMR [400 MHz, DMSO-D6, 25 °C, δ = 2.5 (DMSO)]: δ = 6.03 1H (C**H**_2_=), δ = 5.69 1H (C**H**_2_=), δ = 4.93 1H (COOC**H**(CH_3_)-), δ = 3.65–3.29 18H (-C**H**_2_O(C**H**_2_C**H**(CH_3_)O)_m_-), δ = 3.25 3H (-OC**H**_3_), δ = 1.88 3H (CH_2_=C(C**H**_3_)COO-), δ = 1.16 3H (COOCH(C**H**_3_)-), δ = 1.05 15H (-(CH_2_CH(C**H**_3_)O)_m_-).EPM: ^1^H NMR [400 MHz, DMSO-D6, 25 °C, δ = 2.5 (DMSO)]: δ = 6.03 1H (C**H**_2_=), δ = 5.69 1H (C**H**_2_=), δ = 4.91 1H (COOC**H**(CH_3_)-), δ = 3.65–3.29 18H δ = 3.65–3.29 66H (-C**H**_2_O(C**H**_2_C**H**_2_O)_n_(C**H_2_**C**H**(CH_3_)O)_m_-), δ = 3.25 3H (-OC**H**_3_), δ = 1.88 3H (CH_2_=C(C**H**_3_)COO-), δ = 1.16 3H (COOCH(C**H**_3_)-), δ = 1.05 18H (-(CH_2_CH(C**H**_3_)O)_m_-). Molecular brushes on the base of methacrylic macromonomers (PEM, PM and EPM) were obtained by the conventional free-radical polymerization in a reactor equipped with a stirrer, thermometer, and reflux condenser. Syntheses were carried out in ethyl acetate solutions (30% wt. of the monomers) at temperature 85 °C. The concentration of AIBN was 1.0% wt. with respect to the reaction mixture. After polymerization, the polymers were purified by multiple precipitations from solution by hexane followed by vacuum drying at 50 °C. According to HPLC, the samples contained trace amounts of initial macromonomers and solvents. The 1H NMR spectra are presented in the Supplementary Materials (Figures S1–S3).

The monomers conversion was determined by HPLC (Shimadzu Prominence chromatographic system). During the synthesis, aliquots of the reaction mixture were taken and dissolved by 10 times with ethyl acetate (inhibited by hydroquinone). Amine HPLC column (Kromasil, NH_2_, 4.6 × 250 mm, 5 μm) was used with ethyl acetate as a mobile phase. Molecular weights of polymers were determined by SEC using a Chromos LC-301 instrument with isocratic pump Alpha-10, refractometric detector Waters 410, and two size exclusion columns Phenogel 5u 50A and 10^3^A by Phenomenex (THF was used as a mobile phase, polystyrene standards were used for calibration). A phase transition temperature (T_pt_) was determined from temperature dependence of optical transmittance (colorimeter KFK-2MP with thermostatic cuvette, wavelength of 540 nm).

**Figure 2:**
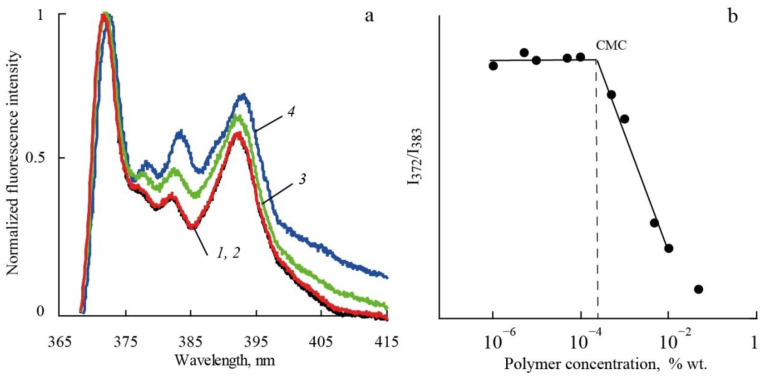
(**a**) Normalized fluorescence spectra of pEPM at concentrations, % wt.: 0.00001 (*1*), 0.0007 (*2*), 0.05 (*3*), 0.1 (*4*); (**b**) I_1_/I_3_ ratio of the vibronic band intensities of pyrene as a function of pEPM concentration at 25 °C.

Critical micelle concentrations (CMC) for polymers in aqueous solutions were determined from measurements of the fluorescence intensity (Shimadzu RF-6000 spectrofluorimeter) of pyrene as a function of polymer concentration in accordance with [21]. Examples of fluorescence spectra obtained are presented in Figure 2a. CMC was determined from dependence of the ratio of the intensities (I_1_/I_3_) of the first (I_1_, 372 nm) and the third (I_3_, 383 nm) vibronic bands of pyrene emission on polymer concentration (Figure 2b).

The interfacial tension at the water–toluene boundary in the presence of the polymers was measured by drop-weight method (temperature 25 °C) [22]. The partition coefficient P is the ratio of the equilibrium concentrations of a polymer in toluene and water (temperature 25 °C). The determination of macromonomer equilibrium concentrations in organic and aqueous phases was performed using size-exclusion chromatography (SEC). Previous to the measurement of the interfacial tensions and distribution coefficients, the solutions of the polymers in the water–toluene system were maintained for two days to reach the equilibrium concentration.

### Methods Molecular Hydrodynamics and Optics

The solution behavior of polymers was studied by the methods of static (SLS) and dynamic light scattering (DLS) using a Photocor Complex instrument (Photocor Complex Inc., Moscow, Russia). The light source was the Photocor-DL diode laser with the wavelength λ = 659.1 nm and controllable power up to 30 mW. The instrument was calibrated using benzene (*R*_V_ = 2.32·10^−5^ cm^−1^). The correlation function of the scattered light intensity was obtained using the Photocor-PC2 correlator with 288 channels and processed using the DynalS software. The measurements were carried out at scattering angles ranging from 45° to 135°. The experiments were performed at 25 °C.

The thermoresponsiveness of synthesized samples in aqueous and buffer solutions investigated were studied using the same Photocor Complex setup, which is equipped with the Photocor-PD detection device for measuring the transmitted light intensity. The characteristics of the equipment and experimental procedure and the conditions for the experiment were previously described in detail [22,23]. The solutions and solvents were filtered into experimental cells. Millipore filters (Millipore Corporation, Burlington, VT, USA) with the pore size of 0.20 μm were used.

**Figure 3:**
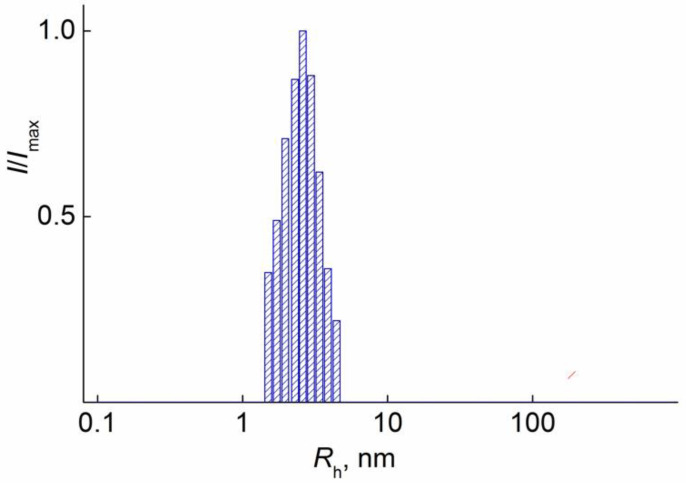
Hydrodynamic radii distribution for solution of pEPM at *c* = 0.0052 g∙cm^−3^ in acetonitrile. *I*_max_ is the maximum intensity of scattered light for given solution concentration.

**Figure 4:**
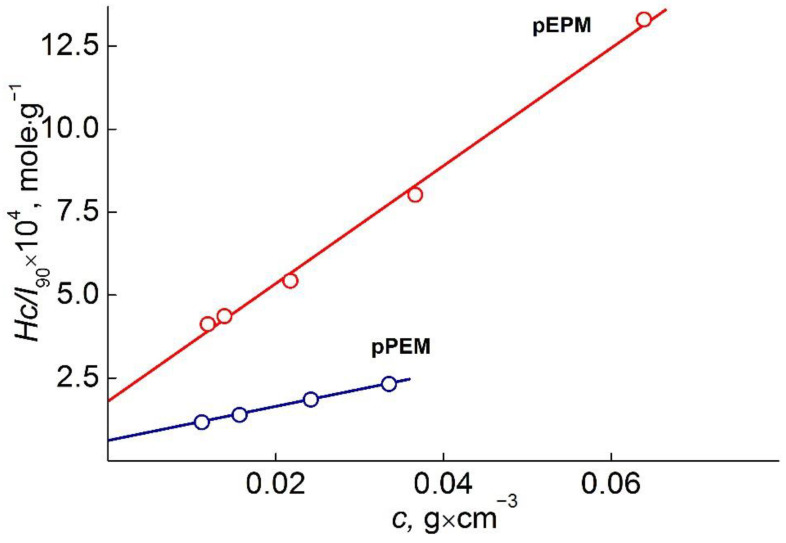
Debye plots for pPEM and pEPM in acetonitrile.

**Figure 5:**
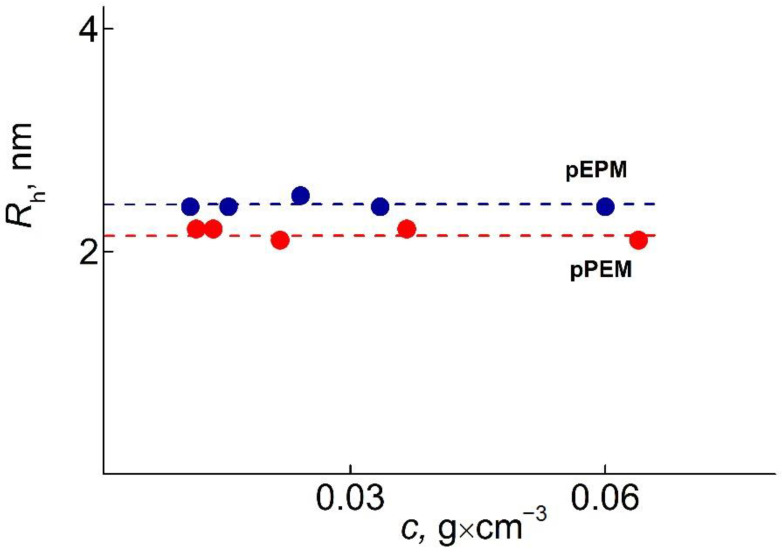
Concentration dependencies of radius R_h-D_ for pEPM and pPEM in acetonitrile.

For all polymer solutions, only one mode was detected (Figure 3). The asymmetry of the light scattering intensity was absent. Therefore, the gyration radii of scattering objects could not be determined, and polymers’ molar masses *M*_w_ were obtained by the Debye method (angle 90°). (Figure 4) [24,25]. The values of the hydrodynamic radii R_h-D_ at given concentration c were determined in the wide concentration range and extrapolated to zero concentration to obtain the hydrodynamic radius R_h-D_ of isolated macromolecules (Figure 5). It should be noted that acetonitrile is a thermodynamically good solvent for the polymers (the positive second virial coefficient).

**Figure 6:**
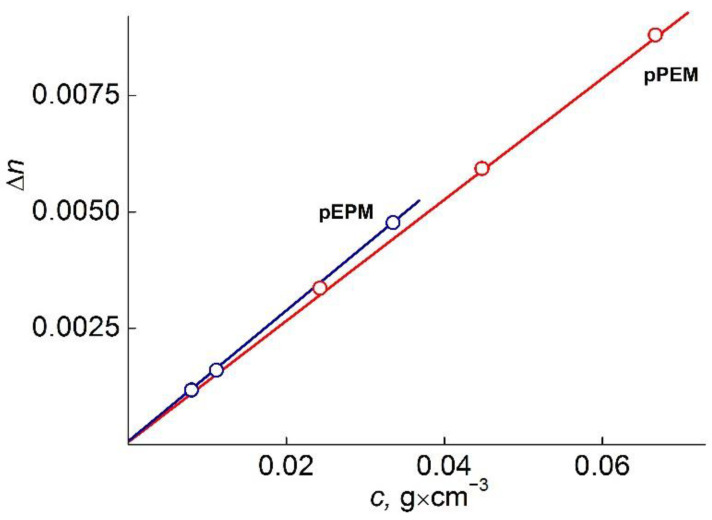
**Dependence** Δ*n* vs. concentration for pEPM and pPEM in acetonitrile.

**Figure 7:**
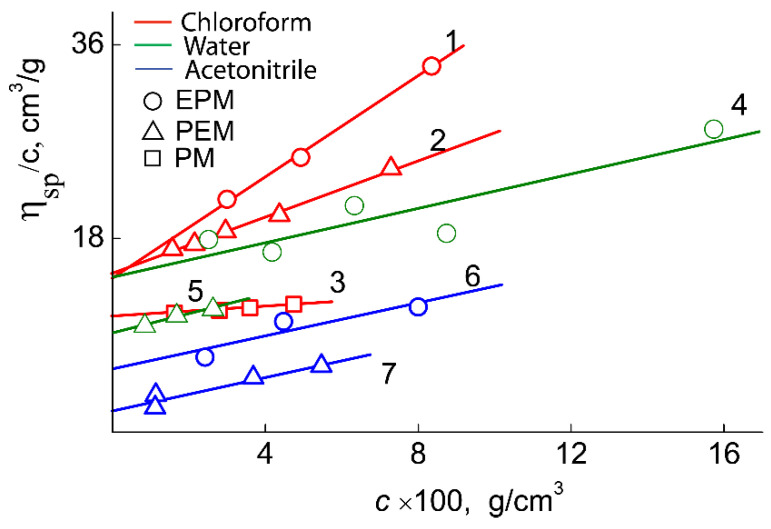
The dependencies η_sp_/c vs. concentration for pEPM in chloroform (1), pPEM in chloroform (2), pPM in chloroform (3), pEPM in water (4), pPEM in water (5), pEPM in acetonitrile (6), pPEM in acetonitrile (7).

The values of refractive index increment *dn/dc* were determined using an RA-620 refractometer (wavelength λ_0_ = 589.3 nm) (Figure 6). The viscometry experiments were carried out on the Ostwald-type Cannon-Manning capillary viscometer (Cannon Instrument Company Inc., State College, PA, USA). The dependencies of the reduced viscosity η_sp_/c on the concentration were analyzed using the equation of Huggins [26] (Figure 7).

η_sp_/*c* = [η] + *k*_H_[η]^2^*c* (1)

where [η] is the intrinsic viscosity and *k*_H_ is the Huggins constant.

## 3. Results and Discussion

**Figure 8:**
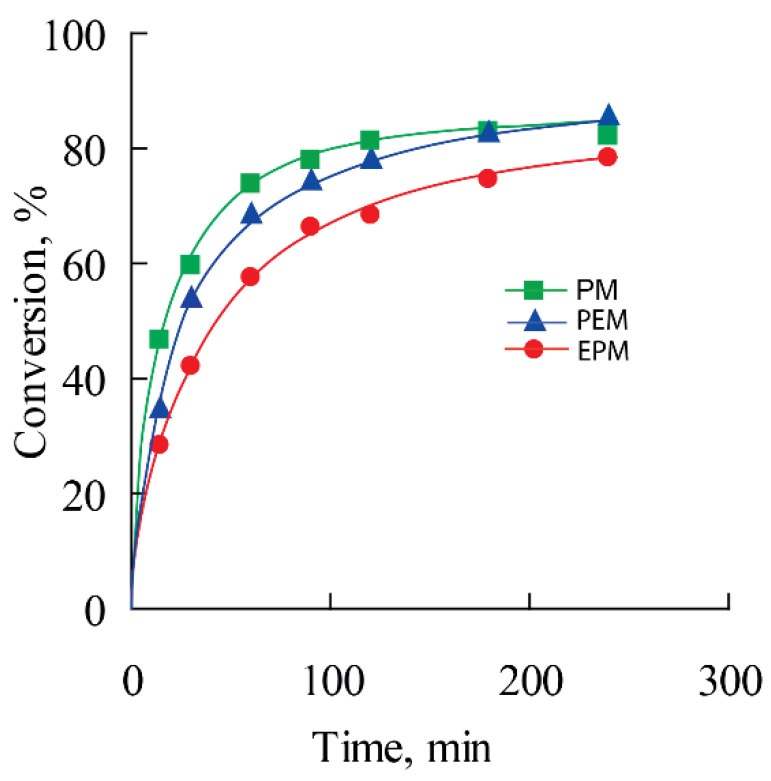
Dependencies of monomer conversion on time: EPM (●), PEM (▲), PM (■).

**Figure 9:**
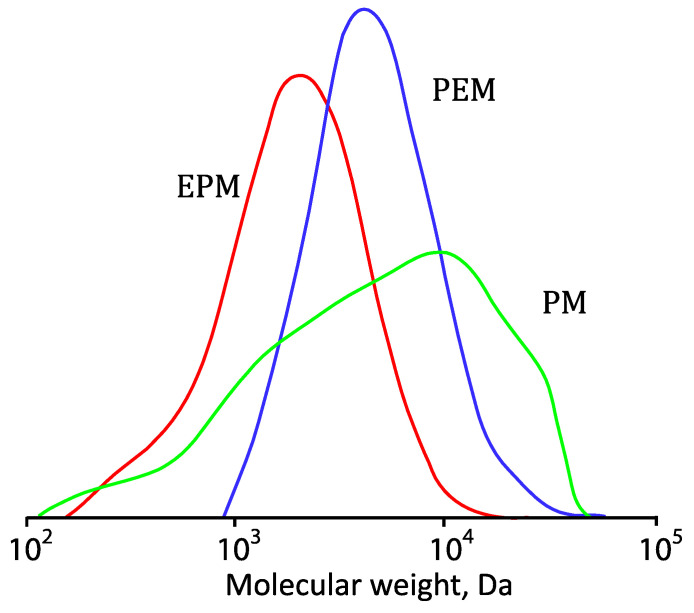
Molecular weights distribution curves of the polymers. An example chromatogram of unpurified pPEM is shown in Figure S4 (Supplementary Materials).

During the synthesis of polymers, the kinetic curves of the consumption of the macromonomers were obtained. Figure 8 shows the dependencies of monomer conversion on synthesis time. All the monomers studied regardless of the substituent composition are actively consumed during polymerization up to a high degree of conversion (80–85%). Molecular weights and molecular weight distribution of the polymers obtained by SEC are shown in Table 1 and Figure 9. The MWD plot for pPM contains an additional shoulder. We believe this is due to the peculiarities of obtaining macromonomers: they may contain traces of dimethacrylates, leading to rare cross-linking of polymer chains.

Table 1 shows the data on the hydrophilic–lipophilic balance (HLB) of monomers, their final conversions (X), and the characteristics of the homopolymers obtained. The HLBs calculated by the Davis method increase logically from 6.9 to 10.2 as the fraction of PO unit in monomers decreases and as the fraction of EO unit increases. The hydrophilic–hydrophobic properties of monomers significantly affect the behavior of homopolymers in water and water–organic systems. It was shown that the most hydrophobic homopolymer pPM is insoluble in water, while the polymers pEPM and pPEM have a limited temperature range of solubility in aqueous media: at a polymer concentration of 1% wt. the phase transition temperatures (Tpt), caused by lower critical solution temperature (LCST), were found to be 64.5 and 40.9 °C, respectively.

**Figure 10:**
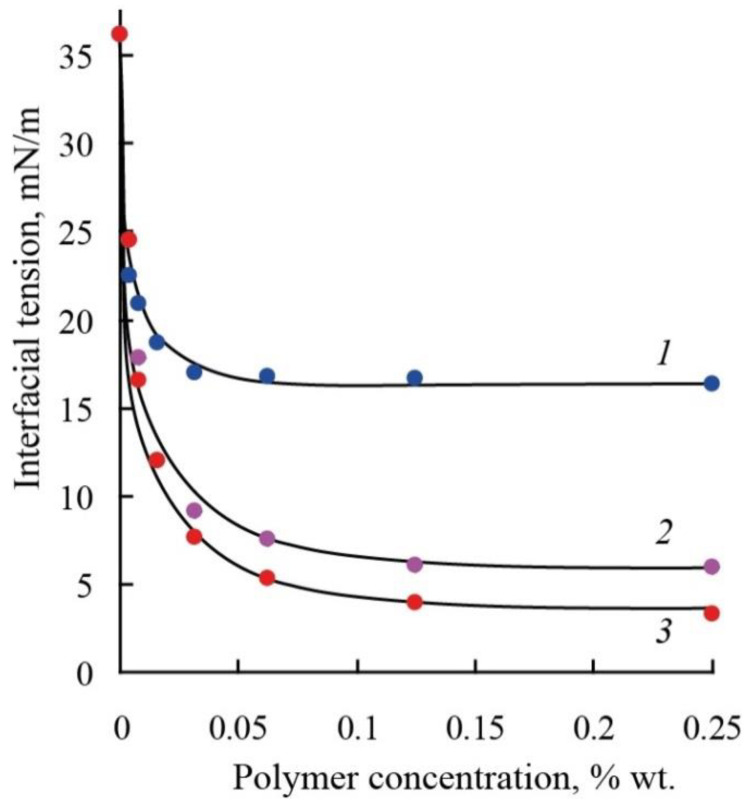
Interfacial tension isotherms for polymers based on macromonomers PM, PEM, and EPM.

Differences in the hydrophilic–hydrophobic properties of the polymers also affect the surface activity of the samples. The study of the interfacial activity of the molecular brushes at the boundary between water and toluene showed (Figure 10) that all the polymers have a pronounced surface activity and intensively decrease the interfacial tension. It was established (Table 1) that an increase in a polymer hydrophilicity leads to an increase in interfacial activity, which is reflected in lower limit values of interfacial tension (plateau region on isotherms of interfacial tension).

The mediated solubility in aqueous media and the high surface activity of pEPM and pPEM polymers can indicate the tendency of polymer macromolecules to associate in water and form multimolecular or monomolecular micelles with a core–shell structure.

As can be seen in Table 2, for pPEM and pEPM, the values of molar mass (MM), intrinsic viscosity [η], and hydrodynamic radius R_h_ determined in different solvents vary strongly. The smallest MM, [η] and R_h_ were obtained in acetonitrile. In this solvent, the characteristic viscosities of both polymers are close to the values [η]_sph_ predicted for solid spherical particles, which, in accordance with the Einstein equation, is defined as [η]_sph_ = 2.5 $\bar{v}$, where $\bar{v}$ is the partial specific volume. For both polymers, $\bar{v}$ = 1.1 cm^3^g^−1^, and, accordingly, [η]_sph_ = 2.7 cm^3^g^−1^, which is close to the experimental values of the intrinsic viscosity of solutions for pPEM and pEPM. This fact and low values of hydrodynamic radii R_h_ suggest that the macromolecules of the studied polymers have a shape close to spherical with a high intramolecular density. In other words, the amphiphilic macromolecules pPEM and pEPM collapse in acetonitrile, which is a selective solvent for polymer blocks. The [η] and R_h_ values for pEPM solutions are higher than those for pPEM. This difference is small, but it may indicate a more compact and dense structure of pPEM molecules, i.e., its greater sensitivity to a selective solvent. Note that the MM of the investigated sample determined by SEC (Table 1) were lower in comparison with molar masses obtained by SLS (Table 2). This fact can be explained as SEC does not give correct information about the MM of polymers with complex architecture [27,28,29,30,31].

Chloroform is also a selective solvent for the studied samples. However, in contrast to acetonitrile, the amphiphilicity of the polymers in this solvent leads to aggregation of molecules, as indicated by higher values of [η] and R_h_. The aggregation degree *m*_a_ is low. Indeed, comparison of MM in chloroform (*M*_chl_) and acetonitrile *M*_ac_ leads to *m*_a_ = *M*_chl_/*M*_ac_ ≈ 4 and 6 for pEPM and pPEM, respectively. In chloroform solutions, the intrinsic viscosity and the hydrodynamic radius characterize the aggregate sizes. The [η] and R_h_ values are not very large, which indicates a high density of the aggregates. In solutions in chloroform, the radii characterizing the sizes of the aggregates and the characteristic viscosities are not very large; this indicates a high density of the aggregates.

In aqueous solutions, it was shown by fluorimetry that the molecular brushes obtained are prone to aggregation. The CMC values for the investigated polymers are presented in Table 1. It was found that polymers have CMC values in the range of a concentration of about 2 mg/L. Accordingly, in chloroform solutions as well as in aqueous solutions, the intrinsic viscosities and the hydrodynamic radii are the characteristics of the aggregates. Note that the values of [η] and R_h_ in water and chloroform differ insignificantly.

**Figure 11:**
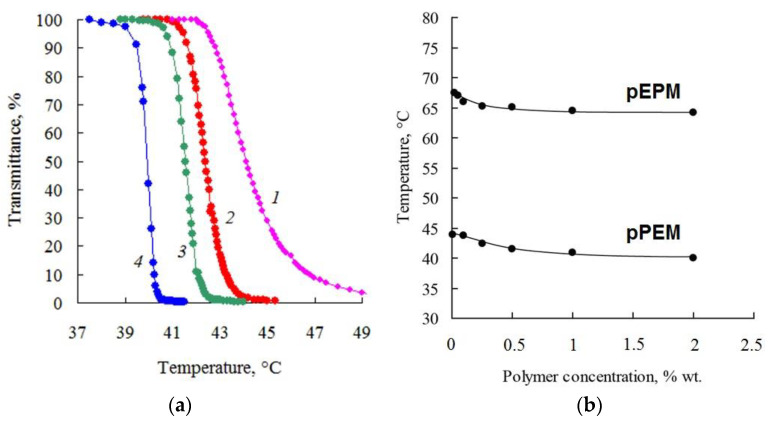
(**a**) Light transmittance vs. temperature at different concentrations of pPEM, %wt.: 0.1 (1), 0.25 (2), 0.5 (3), 2.0 (4); (**b**) Effect of polymer concentration on the Tpt: pEPM (1), pPEM (2).

Figure 11a shows the transmittance values obtained by turbidimetry for aqueous solutions of pPEM as a function of temperature. A sharp decrease in transmittance on heating indicates phase separation in solution. Therefore, the molecular brushes pEPM and pPEM in aqueous solutions are characterized by thermosensitive behavior with lower critical solution temperature (LCST). Figure 11b shows phase diagrams for the investigated polymers. The LCST for more hydrophobic pPEM is about 40 °C, whereas for the more hydrophilic pEPM its value is close to 65 °C.

In addition to the turbidimetry, the solutions of pEPM and pPEM at concentration *c* = 0.005 g·cm^−3^ were investigated by SLS and DLS. The hydrodynamic characteristics of molecular brush aggregates and the temperatures of onset *T*_1_ and finishing *T*_2_ of the phase separation were determined. Note that the *T*_1_ and *T*_2_ values are in good qualitative agreement with the turbidimetry data (Table 3).

**Figure 12:**
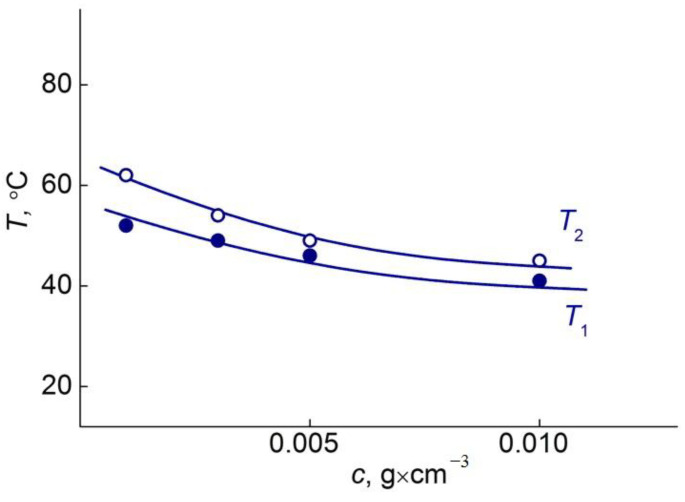
Effect of polymer concentration on the phase separation temperature T_pt_ for pPEM.

This influence is also manifested in the analysis of the dependence of the phase separation temperatures on the polymer concentration and the medium acidity. In water solution, the dependencies of *T*_1_ and *T*_2_ on the concentration (Figure 12) had a character typical for thermoresponsive polymers in the dilute regime [22,23,32].

As can be seen from the Table 3, the difference in behavior of solution of polymers pEPM and pPEM was observed at room temperature. The hydrodynamic radius R_h-rt_ of aggregates at room temperature for pPEM solution was smaller than those for pEPM. Given the MM values, this fact makes it possible to conclude that aggregates of pPEM have high intermolecular density and compact size in comparison with pEPM aggregates. This is also evidenced by the values of intrinsic viscosity (Table 2).

The radii of aggregates in aqueous solutions of pPEM and pEPM increase on heating. Similar behavior was observed earlier for thermo- and pH-responsive polymers with different architecture (linear, copolymers, and brushes) [22,23,32].

It is most interesting to compare the aggregate radii R_h-2_ the obtained for investigated solution at T_2_. It can be seen that radius R_h-2_ for pEPM is two times more than the R_h-2_ for pPEM. That is, the aggregate size in solutions of more hydrophobic pPEM is greater than the aggregate radius of more hydrophilic pEPM at all temperatures. On the other hand, the width of the phase separation interval ΔT = *T*_2_ − *T*_1_ is larger for a more hydrophilic copolymer: ΔT = 3 °C for pPEM and 19 °C for pEPM.

## 4. Conclusions

For homopolymers of oligo (oxyalkylene) methacrylates of three types, differences in behavior in aqueous solutions, organic solvents, and water–toluene binary systems were demonstrated. It was shown that the HLB value of macromonomers plays an important role, which determines such properties of polymers as solubility in water and organic solvents, surface activity at the water–oil interface, as well as thermoresponsive properties. The phase transition temperature of the EPM homopolymer was found in the region of the human body temperature, which, given the tendency of the polymer to form aggregates in aqueous solutions (CMC 2.0 mg/L), opens up possibilities for using the polymer for drug delivery. Molecular brushes were investigated by the methods of molecular hydrodynamics and optics in dilute solutions in acetonitrile, chloroform, and water. The polymers pEPM and pPEM are molecularly dispersedly dissolved in acetonitrile. In these solvents, the studied polymers are characterized by a high intramolecular density, and the shape of their molecules is similar to hard sphere. At the same time, pPEM has a higher intramolecular density. In solutions in chloroform and water, the molecules of both investigated polymers aggregate. In aqueous solutions, pEPM and pPEM exhibit LCST behavior, and the LCST value decreases as the hydrophobicity of the polymer increases.

Classic block copolymers consisting of monomer blocks of different lengths and hydrophobicity are quite well studied and have been shown to be promising for targeted drug delivery, toxicity reduction, targeting, and increasing the therapeutic effectiveness of active pharmaceutical components [9]. The results obtained in this work indicate the high potential of bottlebrush polymers containing oligo(ethylene glycol) and oligo(propylene glycol) blocks. Their main advantages are as follows: simple preparation, ultra-low CMC values, easy adjustment of LCST in a wide range by varying the ratio, length, and mutual arrangement of OEG and OPG blocks in the macromonomer, as well as precise and well-defined length of the side chains in the macromolecule.

## Figures and Tables

**Table 1:** The polymers characteristics.

Sample	X, %	Molecular Weight ^1^			HLB	P	Limiting Interfacial Tension, mN/m	Tpt, °C	CMC, mg/L
		M_n_, kDA	M_w_, kDA	PDI					
pEPM	79.5	7.8	10.8	1.38	10.2	0.06	2.9	64.5	2.9
pPEM	84.5	12.7	17.7	1.39	9.2	0.08	2.0	40.9	2.0
pPM	81.2	10.9	24.3	2.22	6.9	-	-	-	-

^1^ SEC.

**Table 2:** Molar mass and hydrodynamic characteristics of polymers.

Polymers	Solvent	M_w_ × 10^−3^, g·mol^−1^	dn/dc cm^3^·g^−1^	*(*R_h-D_)*,* R_h-m_, nm	[η], cm^3^·g^−1^
pEPM	Water	70	0.15	4.2	14
	Chloroform	100	0.03	4.2	15
	Acetonitrile	22	0.14	2.4	5
pPEM	Water	200	0.14	4.2	9
	Chloroform	250	0.03	4.2	15
	Acetonitrile	39	0.13	2.1	2
pPM	Chloroform	27	0.03	4.9	11

**Table 3:** The phase separation temperatures and hydrodynamic characteristics of aggregates of polymer solution at concentration = 0.005 g∙cm^−3^.

Polymers	Solvent	R_h-rt_, nm at r. t	*T*_1_, °C	*T*_2_, °C	*T*_1_, °C	*T*_2_, °C	R_h-m_, nm at *T*_1_	R_h-m_, nm at *T*_2_
pEPM	Water	5.8	48	67	48	67	135	800
pPEM	Water	3.3	46	49	46	49	420	450

R_h-rt_, nm size of aggregates in water solutions at room temperatures.

## Data Availability

The data presented in this study are available upon request from the corresponding author.

## Supplementary Materials

The following supporting information can be downloaded at: https://www.mdpi.com/article/10.3390/polym14245556/s1, Figure S1: ^1^H NMR spectrum of pPM; Figure S2: ^1^H NMR spectrum of pEPM; Figure S3. ^1^H NMR spectrum of pPEM; Figure S4: SEC plot for pPEM final reaction mixture.
