# Supplementary material for: The Solution Properties of Polymethacrylate Molecular Brushes with Oligo(ethylene glycol) and Oligo(propylene glycol) Side Chains
Source: Polymers (Basel). 2022 Dec 19;14(24):5556. doi: 10.3390/polym14245556 (PMC9786129; doi:10.3390/polym14245556)
Supplement: Supplementary file 1 [file polymers-14-05556-s001.zip › polymers-2095194-supplementary.pdf]

Supplementary Information for:

The Solution Properties of Polymethacrylate Molecular Brushes with Oligo(ethylene glycol) and Oligo(propylene glycol) Side Chains

Maria Simonova[1,*], Alexander Simagin[2], Denis Kamorin[2], Sergey Orekhov[2], Alexander Filippov[1], and Oleg Kazantsev[2]

[1] Institute of Macromolecular Compounds of the Russian Academy of Sciences, Bolshoy Prospekt 31, 199004 Saint Petersburg, Russia

[2] Nizhny Novgorod State Technical University n.a. R.E. Alekseev, 24 Minin Street, 603950 Nizhny Novgorod, Russia

The Figures S1-S3 show the spectra of the purified polymers. As can be seen from Figure S1, the purified polymers contain very few residual monomers. Their content can be approximately estimated based on the integral intensity of the signals for the vinyl protons (m) of the monomer and the methine group (c) in the polymer:

Resid. mon.=m/(m+c)= 1/(1+155.99)=0.006 (0.6%).

Fig. S1. ^{1}H NMR spectrum of pPM

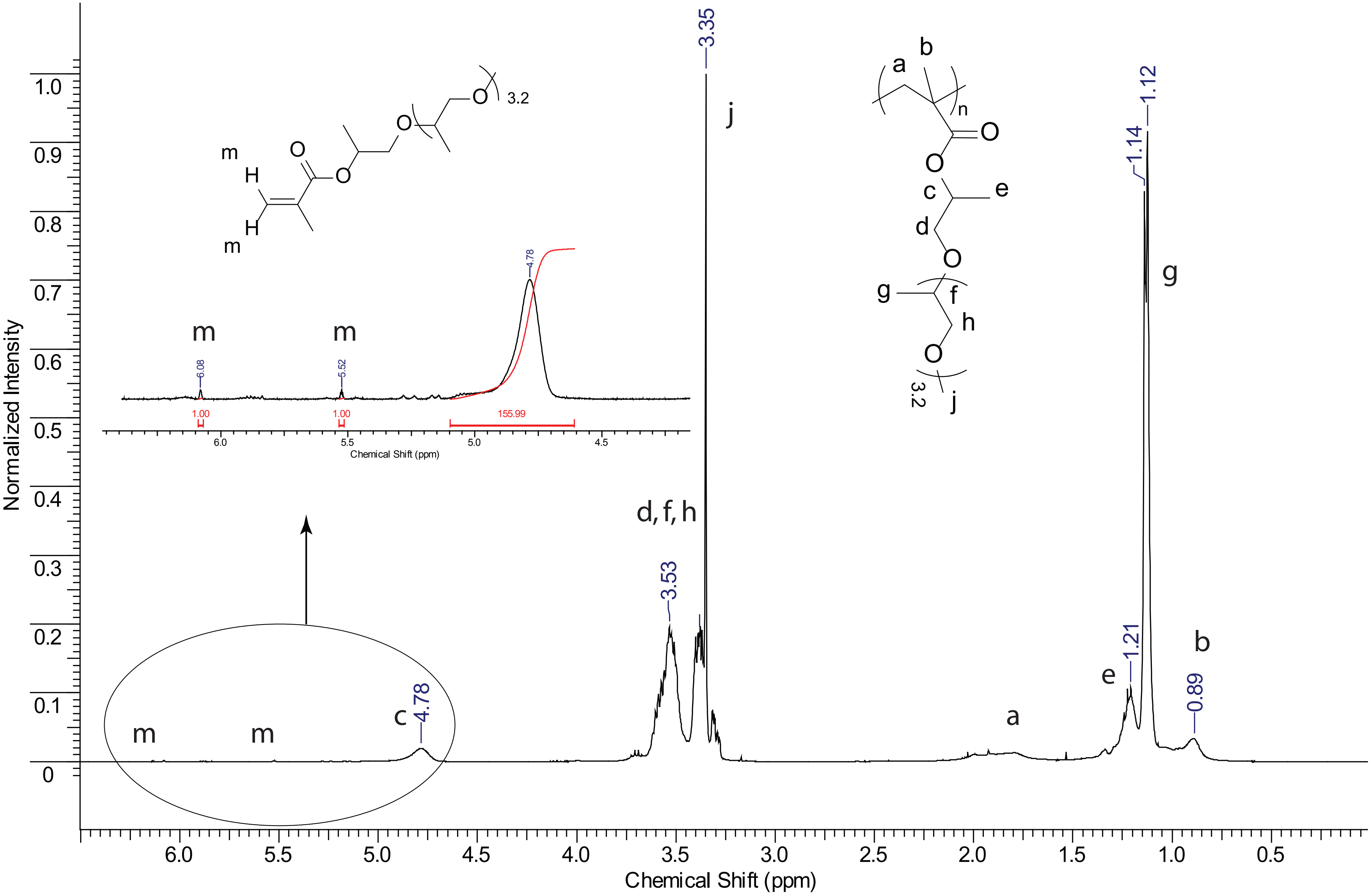

Fig. S2. ^{1}H NMR spectrum of pEPM

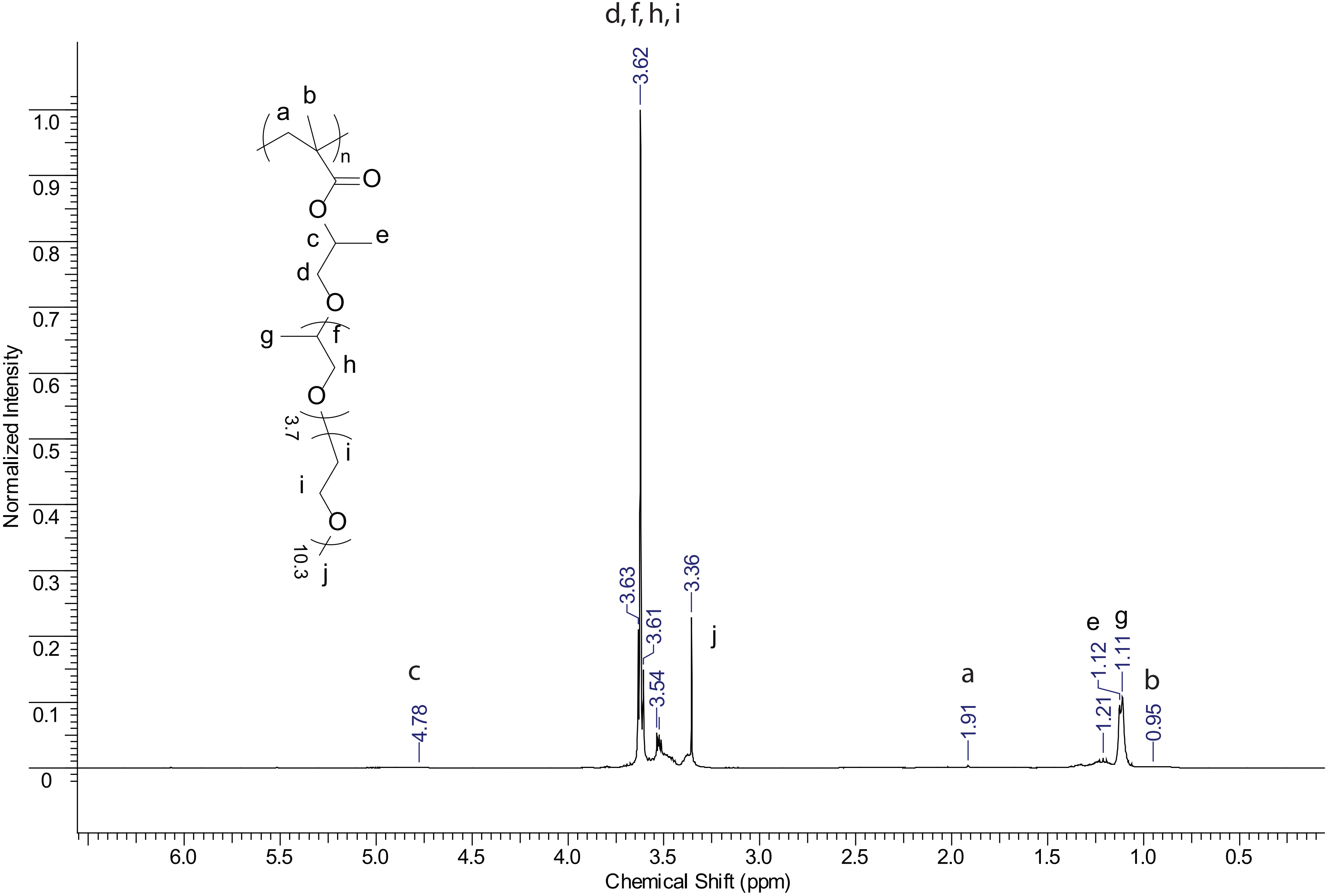

Fig. S3. ^{1}H NMR spectrum of pPEM

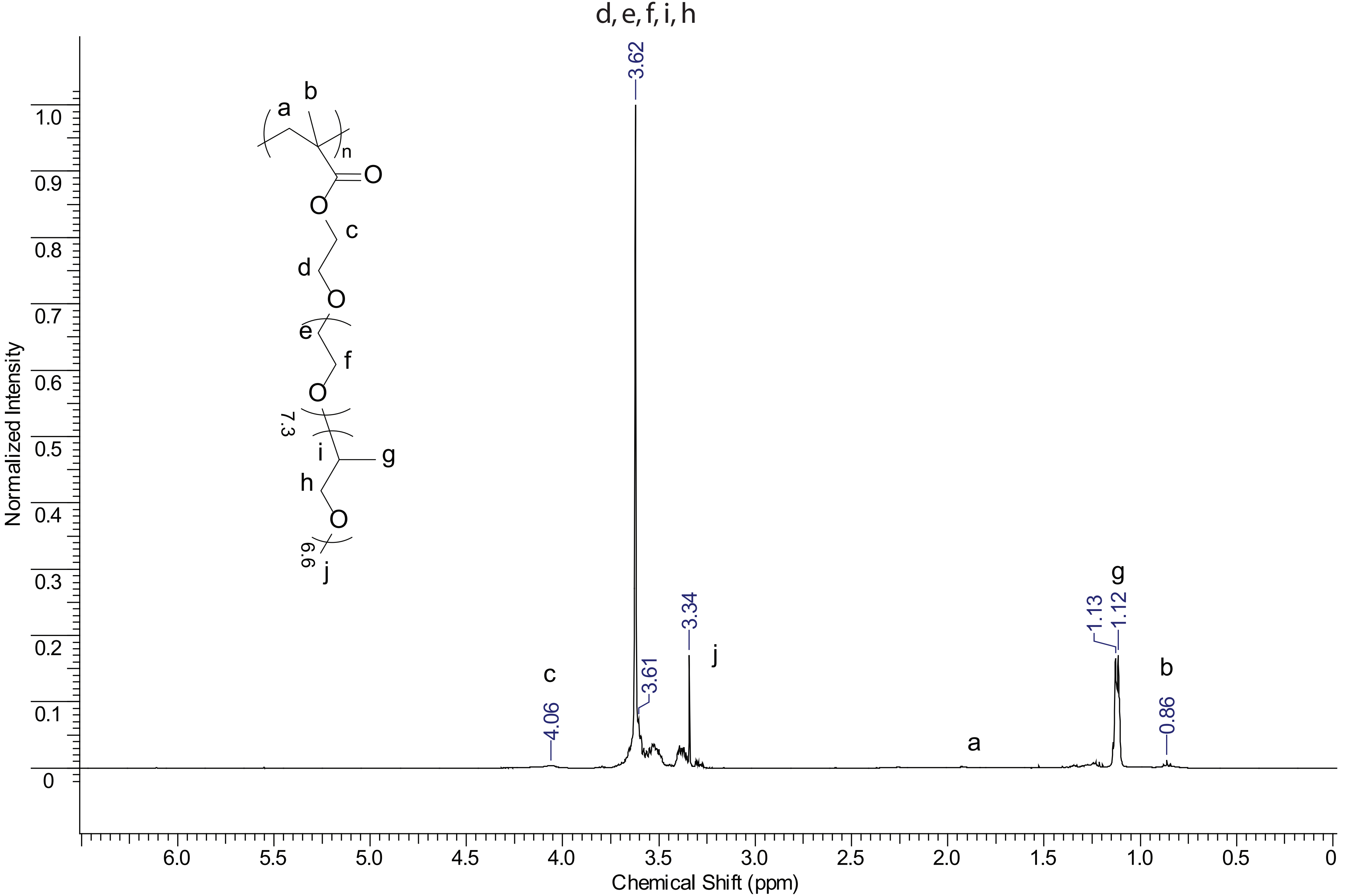

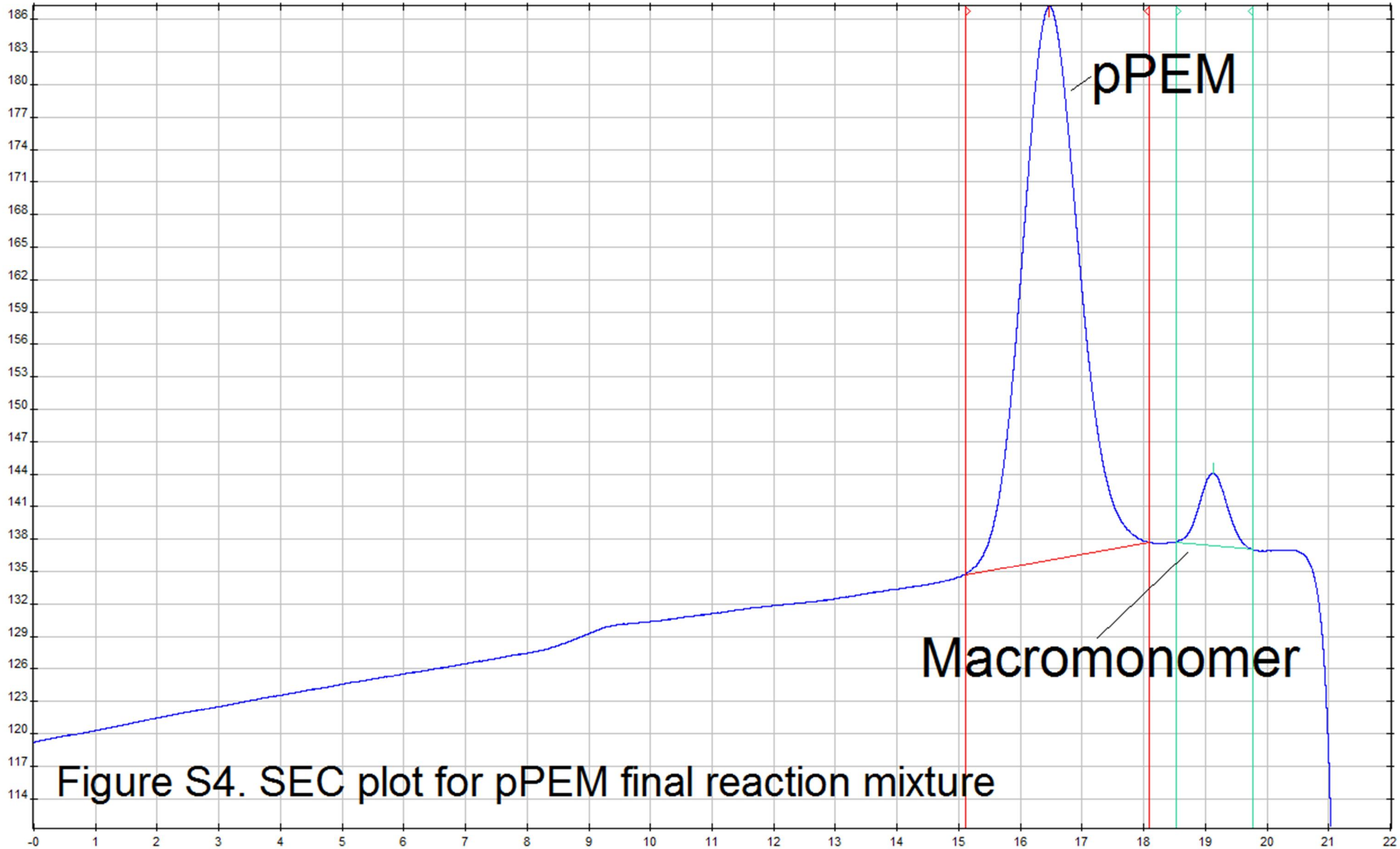

Figure S4. SEC plot for pPEM final reaction mixture
